# Application of Nanostructures in Electrochromic Materials and Devices: Recent Progress

**DOI:** 10.3390/ma3125029

**Published:** 2010-11-26

**Authors:** Jinmin Wang, Xiao Wei Sun, Zhihui Jiao

**Affiliations:** 1 School of Electrical and Electronic Engineering, Nanyang Technological University, 50 Nanyang Avenue, Singapore 639798, Singapore; E-Mails: jmwang@ntu.edu.sg (J.W.); JIAO0013@e.ntu.edu.sg (Z.J.); 2 Department of Applied Physics, College of Science, Tianjin University, Tianjin 300072, China

**Keywords:** electrochromic, ZnO nanowire, WO_3_, TiO_2_, Prussian blue, crystalline, mesoporous, nanostructures

## Abstract

The recent progress in application of nanostructures in electrochromic materials and devices is reviewed. ZnO nanowire array modified by viologen and WO_3_, crystalline WO_3_ nanoparticles and nanorods, mesoporous WO_3_ and TiO_2_, poly(3,4-ethylenedioxythiophene) nanotubes, Prussian blue nanoinks and nanostructures in switchable mirrors are reviewed. The electrochromic properties were significantly enhanced by applying nanostructures, resulting in faster switching responses, higher stability and higher optical contrast. A perspective on the development trends in electrochromic materials and devices is also proposed.

## 1. Introduction

Electrochromism, a reversible change in a material’s optical properties (transmittance, absorbance and reflectance) under an applied voltage [1,2,3], is an old phenomenon which was discovered 40 years ago [4]. Since its discovery, considerable progress has been achieved in the syntheses of electrochromic (EC) materials, the fabrications of EC devices, the improvements of EC properties and the applications of EC materials that have been extended to smart windows, displays, antiglare mirrors and active camouflages [5,6,7,8,9,10,11,12]. Among them, smart windows represent an important application because they can effectively save energy by regulating solar heat gain, and provide indoor comfort by reversible color changes. EC smart windows have become more and more significant because the warming climate and energy crisis require a marked and substantial energy-saving to combat conventional energy source consumption.

Many inorganic and organic materials exhibit EC properties. Common inorganic EC materials include WO_3_ [13], NiO [14], TiO_2_ [15], V_2_O_5_ [16] and PB [17]; while viologens [18], polyaniline (PANI) [19], poly(3,4-ethylenedioxythiophene) (PEDOTs) [20] are common organic and polymer EC materials. For the inorganic EC materials, WO_3_, TiO_2_, V_2_O_5_ films show cathodic coloration states under negative potentials and bleaching states under positive potentials; the coloration/bleaching of these EC materials results from the insertion/extraction of electrons and charge balancing ions (H^+^, Li^+^, Na^+^, K^+^ ions) accompanied by the reduction/oxidation reactions [21]. In contrast, nickel oxide [22] and iridium oxide [23,24] show anodic coloration under a positive potential because their reduced states are colorless and oxidized states are colored. Prussian blue (PB), with a blue color in its original state, can be bleached to a colorless state (reduced state) under a negative potential and can be recovered to its coloration state (oxidized state) under a positive potential [25].

Electrochromism involves the ions insertion/extraction into/out of EC materials, so nanostructures with small sizes and large specific surface areas, are expected to facilitate the ion insertion/extraction process, and then to enhance the properties of EC materials and devices. EC materials, devices and their applications have been well reviewed by experts in the field, especially by Granqvist [26,27,28,29,30,31] and Deb [3]. In this review, we emphasize recent progress in applying nanostructures in EC materials and devices. Some interesting nanostructures, including ZnO nanorod array, crystalline WO_3_ nanoparticles and nanorods, mesoporous WO_3_, mesoporous TiO_2_, PEDOT nanotubes prepared from anodic aluminum oxide (AAO) template, V_2_O_5_ nanowires, PB nanoinks and nanostructures in switchable mirrors, are briefly reviewed and a perspective is proposed.

## 2. Nanostructures in EC Materials and Devices

### 2.1. ZnO Nanowire Array

ZnO nanowire arrays have been fabricated by both gas phase and solution phase methods. Their applications were mostly focused on photoluminescence, electroluminescence, gas sensors and transistors. Recently, Vayssieres [32] reported a simple aqueous thermal decomposition method to fabricate uniform and large-area ZnO nanowire arrays on indium tin oxide (ITO) coated glass at a low temperature (<100 °C), which does not affect the conductivity of the substrate.

**Figure 1:**
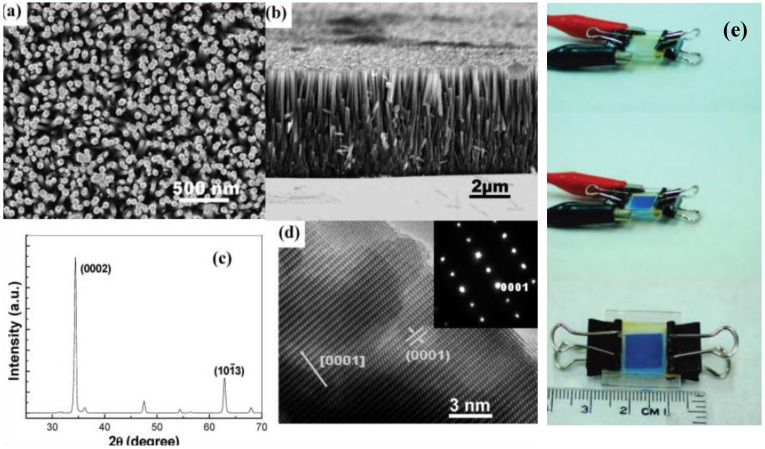
(a), (b) Scanning electron microscopy (SEM) images, (c) X-ray powder diffraction (XRD) pattern, (d) high-resolution transmission electron microscope (HRTEM) image of the ZnO nanowire array hydrothermally grown on ITO coated glass and (e) photos of the EC device showing different colors at different voltages. Reproduced with permission from Ref. [33]; Copyright 2008 American Chemical Society.

**Figure 2:**
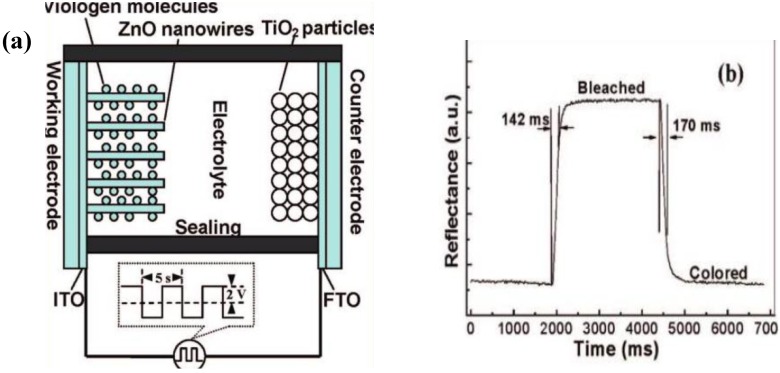
(a) Schematic illustration of the ZnO nanowire EC device and (b) plot of the reflectance of ZnO EC device *versus* time. Reproduced with permission from Ref. [33]; Copyright 2008 American Chemical Society.

**Figure 3:**
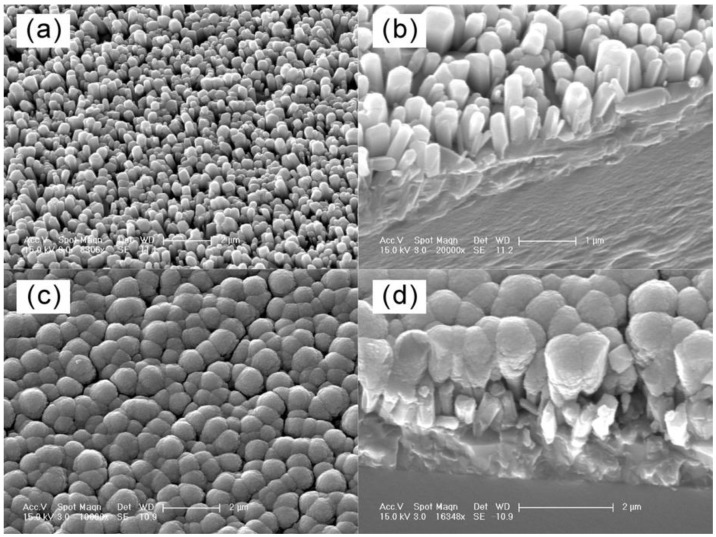
SEM images of (a), (b) the ZnO nanorod arrays, (c) and (d) WO_3_-modified ZnO nanorod arrays. Reproduced with permission from Ref. [34]; Copyright 2009 IOP Publishing Ltd.

Sun and Wang [33] successfully applied ZnO nanowire array in EC device and fabricated a fast-switching display (Figure 1). ZnO nanowire array was modified with viologen molecules which act as the EC material (Figure 2a). The ZnO nanowire electrochromic device exhibited fast switching time (170 and 142 ms for coloration and bleaching, respectively, which is faster than those of the EC devices based on viologen molecules. The coloration time is defined as the time it takes for the reflectance to decrease by 2/3 of the reflectance difference between the steady bleached and the colored state.), high coloration efficiency (196 C^−1^ cm^2^) and good stability (Figure 3). The improved EC properties of the ZnO nanowires EC device is attributed to the large surface area, well-crystalline structure and good electron transport properties of the ZnO nanowire array. The fabricated ZnO nanowire electrochromic device is promising for application in low-cost “electronic papers” (e-papers).

**Figure 4:**
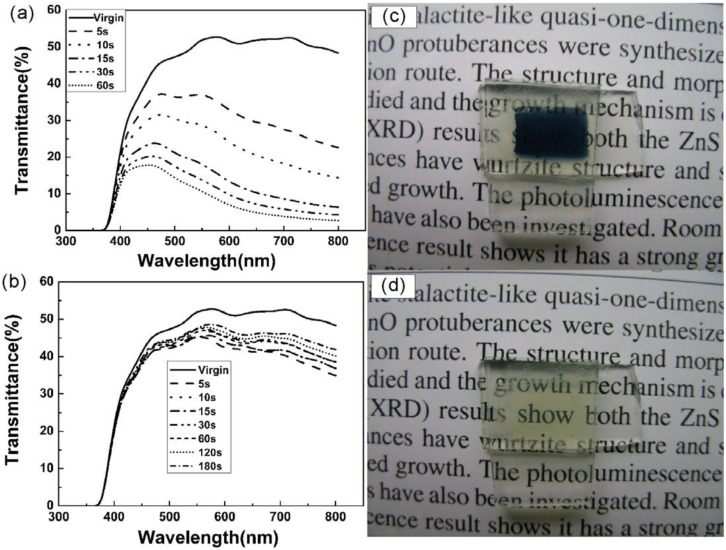
Transmittance spectra of the WO_3_-modified ZnO nanorod arrays EC device at the original state and −3 V potential (a) colored for 5, 10, 15, 30 and 60 s, (b) bleached for 5, 10, 15, 30, 60, 120 and 180 s, (c) colored state and (d) bleached state. Reproduced with permission from Ref. [34]; Copyright 2009 IOP Publishing Ltd.

Enlightened by Sun and Wang’s work, Wang *et al.* [34] fabricated WO_3_ nanoparticle-modified ZnO nanorod arrays electrode. Amorphous WO_3_ was grown on the surface of ZnO nanorod arrays (Figure 3) by pulsed laser deposition (PLD). The EC device also shows high electrochromic stability and fast switching speed (Figure 4).

### 2.2. Crystalline WO_3_ Nanostructures

Most of the EC investigations on WO_3_ were focused on its amorphous films [35,36,37]. Compared with the amorphous structure, crystalline WO_3_ is much more stable due to the denser structure and slower dissolution rate in electrolytes. However, crystalline WO_3_ bulk material usually has slow switching response. To improve the switching response, nanocrystalline WO_3_ were applied in EC materials and devices in recent years.

**Figure 5:**
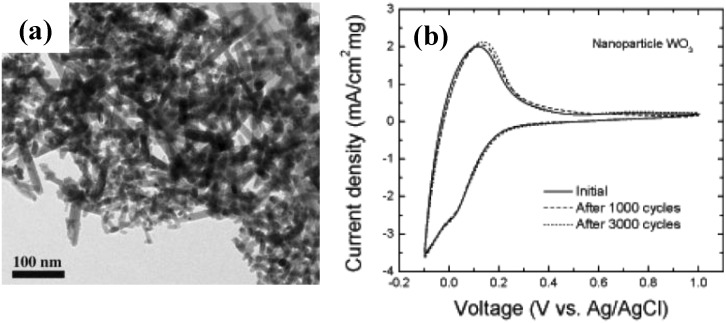
(a) TEM image of crystalline WO_3_ nanoparticles and nanorods prepared by hot-wire chemical vapor deposition and (b) cyclic voltammetry (CV) curves of the EC film showing the high cycling stability. Reproduced with permission from Ref. [38]; Copyright 2006 Wiley-VCH Verlag GmbH & Co. KGaA.

Dillon *et al*. [38] has synthesized crystalline WO_3_ nanoparticles and nanorods (Figure 5a) by hot-wire chemical vapor deposition and fabricated an electrochromic film by electrophoresis deposition. The fabricated crystalline WO_3_ nanoparticle film has greater charge density for H^+^ ions intercalation, which is attributed to a larger active surface area and the low density of the films. Compared with amorphous WO_3_ film, the crystalline WO_3_ nanoparticle film exhibited better cycling stability in H_2_SO_4_ solution (Figure 5b).

**Figure 6:**
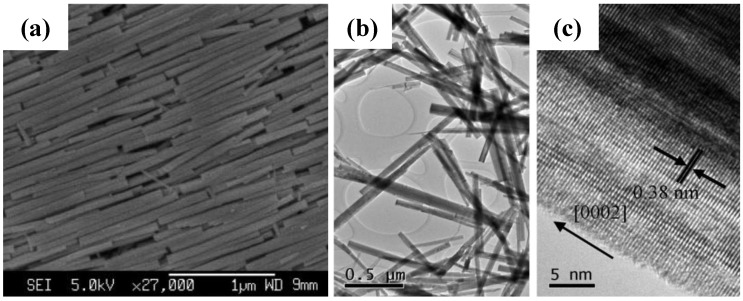
(a) FESEM image, (b) TEM image and (c) HRTEM image of uniform crystalline WO_3_ nanorods. Figure 6a shows the assembly of WO_3_ nanorods. Reproduced with permission from Ref. [39]; Copyright 2008 American Chemical Society.

**Figure 7:**
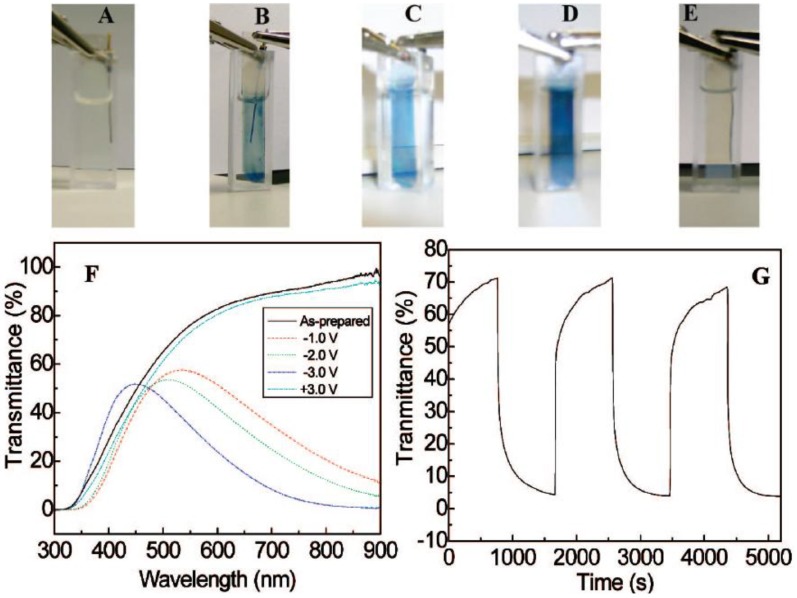
(A)–(E) Switching characteristics of the WO_3_ nanorod film, (F) UV-vis spectra at different voltages and (G) *in situ* coloration/bleaching characteristic showing the switching responses. Reproduced with permission from Ref. [39]; Copyright 2008 American Chemical Society.

**Figure 8:**
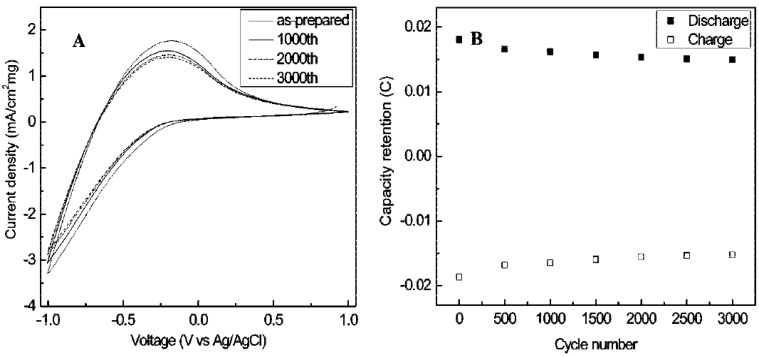
(A) CV curves and (B) capacity retention of the WO_3_ nanorods film showing the high stability. Reproduced with permission from Ref. [39]; Copyright 2008 American Chemical Society.

Wang *et al*. [39,40] hydrothermally synthesized uniform crystalline WO_3_ nanorods (Figure 6) with NaCl as the capping agent firstly, and fabricated a transparent EC film by a drop-casting process. The synthesized WO_3_ nanorods were assembled on the surface of the film, showing tunable coloration (green, green-blue, and blue colors) at different voltages (Figure 7), high stability (Figure 8) both in LiClO_4_ and H_2_SO_4_ electrolytes, comparable contrast and switching coloration/bleaching responses. The assembly of WO_3_ nanorods allows sufficient Li^+^ ions to be intercalated into WO_3_ nanorods, resulting in a high contrast. The displayed colors of the WO_3_ nanorod film can be tuned by changing the applied voltages to control the amount of intercalated Li^+^ ions into WO_3_ nanorods. The crystalline structure of the synthesized WO_3_ nanorods greatly enhances the stability of the EC film. The coarse surfaces of nanorods and interstices among nanorods increase the specific surface area of the film and accelerate the intercalation/deintercalation of Li^+^ ions, resulting in fast coloration/bleaching switching. The WO_3_ nanorod film can be used in H_2_SO_4_ electrolyte due to the crystalline structure. Compared with the EC switching characteristics of WO_3_ nanorod film in lithium-based electrolyte, faster response, much higher charge density, comparable coloration efficiency and contrast were achieved in H_2_SO_4_ electrolyte.

**Figure 9:**
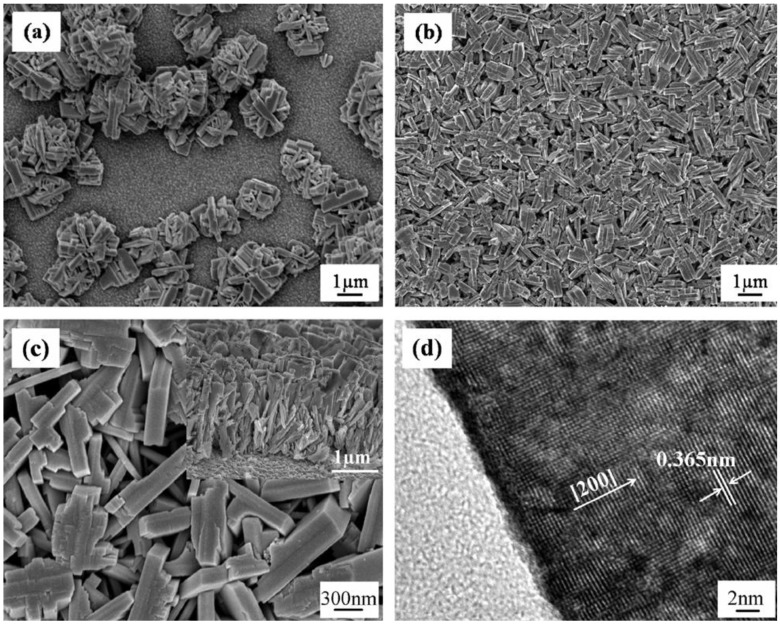
(a) SEM images of WO_3_ microparticles grown without a seed layer; (b), (c) as-prepared WO_3_ thin film and (d) HRTEM image of a single WO_3_ plate-like nanostructure. Inset in Figure 9c: a cross sectional SEM image. Reproduced with permission from Ref. [41]; Copyright 2010 IOP Publishing Ltd.

**Figure 10:**
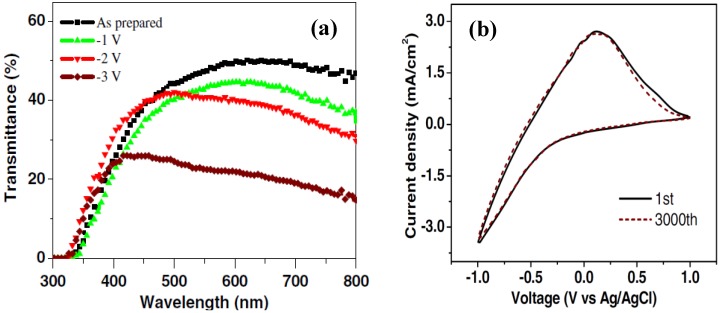
(a) Transmittance spectra measured at different voltages and (b) CV curves of the hydrothermally grown WO_3_ thin film. Reproduced with permission from Ref. [41]; Copyright 2010 IOP Publishing Ltd.

**Figure 11:**
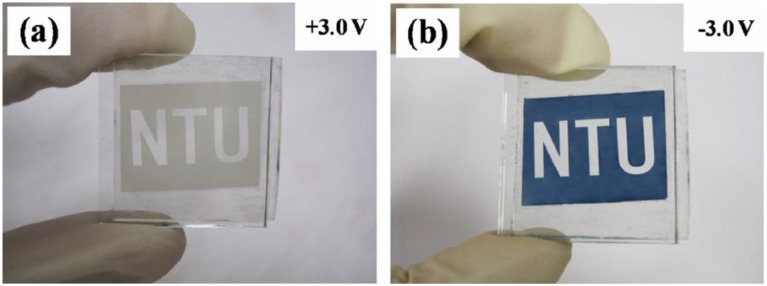
An EC display based on the hydrothermally grown WO_3_ thin film. Reproduced with permission from Ref. [41]; Copyright 2010 IOP Publishing Ltd.

Recently, Jiao *et al*. [41] prepared crystalline plate-like WO_3_ nanostructures (Figure 9) on fluorine-doped tin oxide (FTO) coated glass by a crystal-seed-assisted hydrothermal method. The hydrothermally grown film is well adhesive to the substrate, which is attributed to the use of crystal seeds. The film, consisting of crystalline WO_3_ nanostructures, exhibited tunable transmittance modulation (Figure 10a) under different voltages, high cycling stability (Figure 10b), and high optical contrast (Figure 11).

According to the above results, we draw a conclusion that crystalline WO_3_ nanostructures improve the cycling stability (due to the crystalline structure) of the EC materials and devices without degrading the switching responses, contrast and coloration efficiency (due to the nanoscale structure). This is important progress towards the practical application of high-performance EC devices.

### 2.3. Mesoporous WO_3_

**Figure 12:**
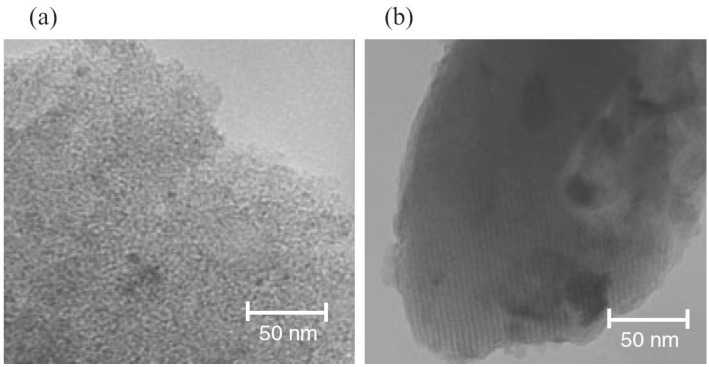
TEM images of tungsten oxide films (a) deposited at −0.2 V, yielding a wormhole-like structure, (b) deposited at −0.5 V, resulting in a lamellar structure. Reproduced with permission from Ref. [42]; Copyright 2003 Wiley-VCH Verlag GmbH & Co. KGaA.

**Figure 13:**
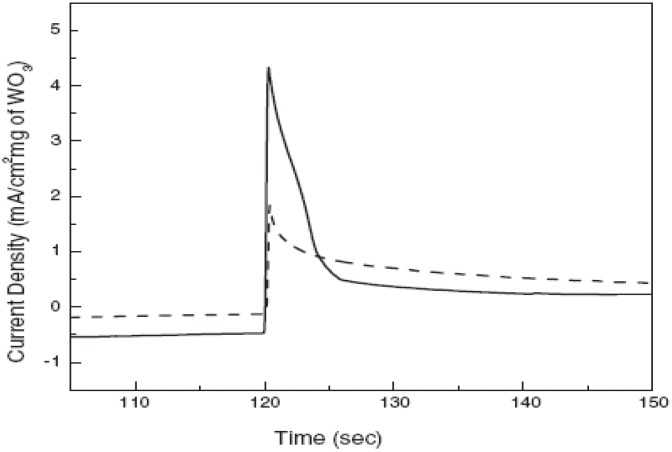
Chronoamperometry with voltage step from −0.5 to + 0.5 V (solid line: lamellar phase mesoporous tungsten oxide, dashed line: control film). Reproduced with permission from Ref. [42]; Copyright 2003 Wiley-VCH Verlag GmbH & Co. KGaA.

Mesoporous EC materials have quite thin walls and large specific surface areas, which makes them interesting for enhancing EC properties. Baeck *et al*. [42] have successfully synthesized mesoporous tungsten oxide films with lamellar structure (Figure 12) by electrodeposition using a templating agent. Lamellar mesoporous tungsten oxide shows greater current density for hydrogen intercalation and faster switching responses (Figure 13) compared to nonporous structures. The functional improvements may be due to the larger surface area of mesoporous tungsten and facilitated charge transport.

**Figure 14:**
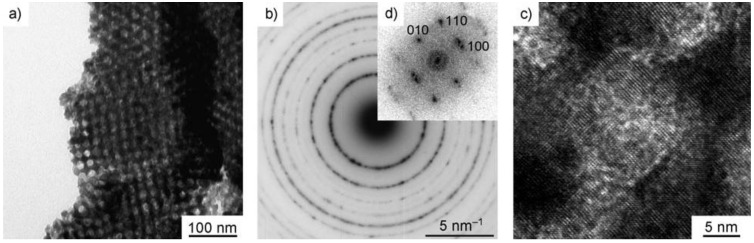
(a) TEM image, (b) Selected area electron diffraction (SAED) pattern and (c) HRTEM image of the mesoporous ordered WO_3_ network after crystallization. (d) Fourier transform of the image shown in (c). Reproduced with permission, Ref. [43]; Copyright 2006 Wiley-VCH Verlag GmbH & Co. KGaA.

**Figure 15:**
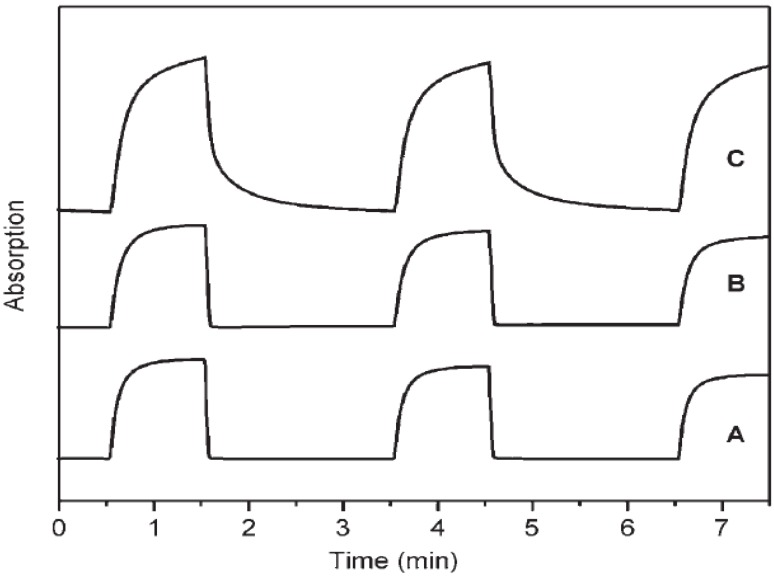
Coloration-bleaching characteristics of mesoporous WO_3_ films at a wavelength of 630 nm and at voltage steps of ±1 V. Reproduced with permission from Ref. [43]; Copyright 2006 Wiley-VCH Verlag GmbH & Co. KGaA.

**Figure 16:**
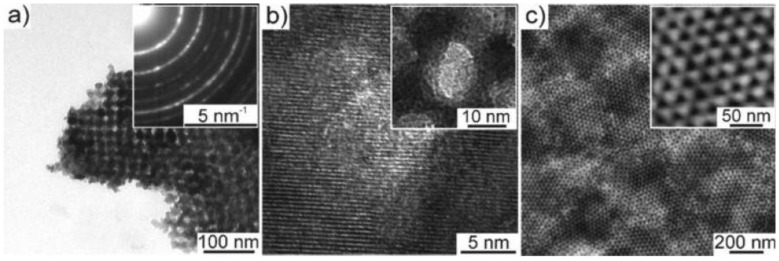
(a), (b) TEM images and (c) tapping mode AFM images of periodically ordered WO_3_ thin films after crystallization at 550 °C showing the homogeneity of the mesoporous structure. Reproduced with permission from Ref. [44]; Copyright 2007 American Chemical Society.

Brezesinski *et al*. [43] synthesized mesoporous WO_3_ EC thin film (Figure 14) by evaporation-induced self-assembly using a block-copolymer template. The three-dimensional mesoporosity significantly improves the electrochromic response times, because of shorter diffusion pathlengths compared to the dense materials (Figure 15). The combination of mesoporosity and crystallinity leads to improved reversibility of ion insertion/extraction and cycling stability. Amorphous mesoporous WO_3_ films suffer from irreversible degradation because of their structural modifications. By annealing at 550 °C in air, fully crystalline mesoporous WO_3_ thin films are obtained. Sallard *et al*. [44] also prepared crystalline mesoporous WO_3_ films (Figure 16) by annealing amorphous mesoporous WO_3_ films. The crystallinity degree ranging from fully amorphous to crystalline can be adjusted without mesostructural collapse. Electrochemical results show that only the highly crystalline mesoporous WO_3_ films exhibit long-term EC stability.

Though mesoporous WO_3_ films have faster responses due to the large specific surface area, the EC stability is dependent on the crystallinity of the mesoporous structure. The film coating process using the WO_3_ mesoporous structures also needs to be developed to achieve good-adhesion EC films on transparent substrates.

### 2.4. Mesoporous TiO_2_

EC device based on dye-modified semiconductor electrodes has much improved switching response and enhanced contrast, especially for displays such as electronic paper and billboards [45,46,47,48,49]. The key part of these devices is a working electrode composed of a nanocrystalline semiconductor modified with electrochromophoric molecular species, such as redox active viologen derivatives. TiO_2_ nanostructures have been successfully used for this purpose, resulting in high-performance EC devices.

**Figure 17:**
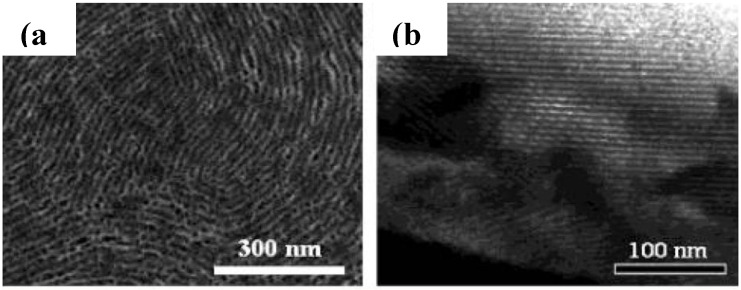
(a) SEM image and (b) TEM image for mesoporous TiO_2_ film after calcination at 400 °C for 4 h. Reproduced with permission from Ref. [50]; Copyright 2004 American Chemical Society.

**Figure 18:**
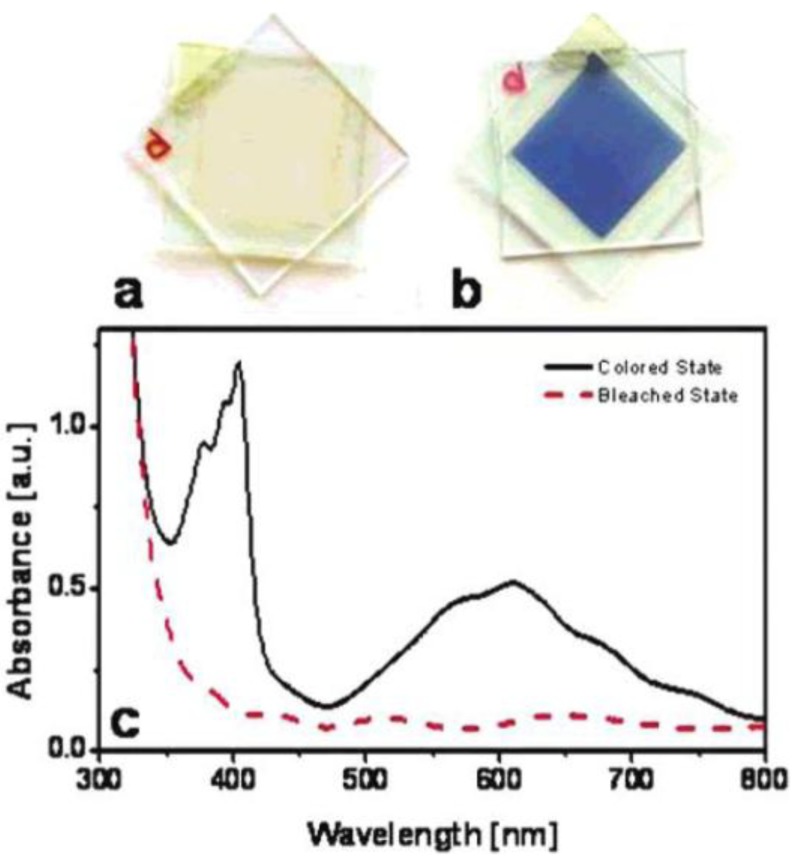
Photos of the mesoporous TiO_2_ film EC cell before (a) and after (b) applying −2.5 V, and UV-vis absorption spectra (c) of the same samples before (dashed line) and after (solid line) applying −2.5 V. Reproduced with permission from Ref. [50]; Copyright 2004 American Chemical Society.

**Figure 19:**
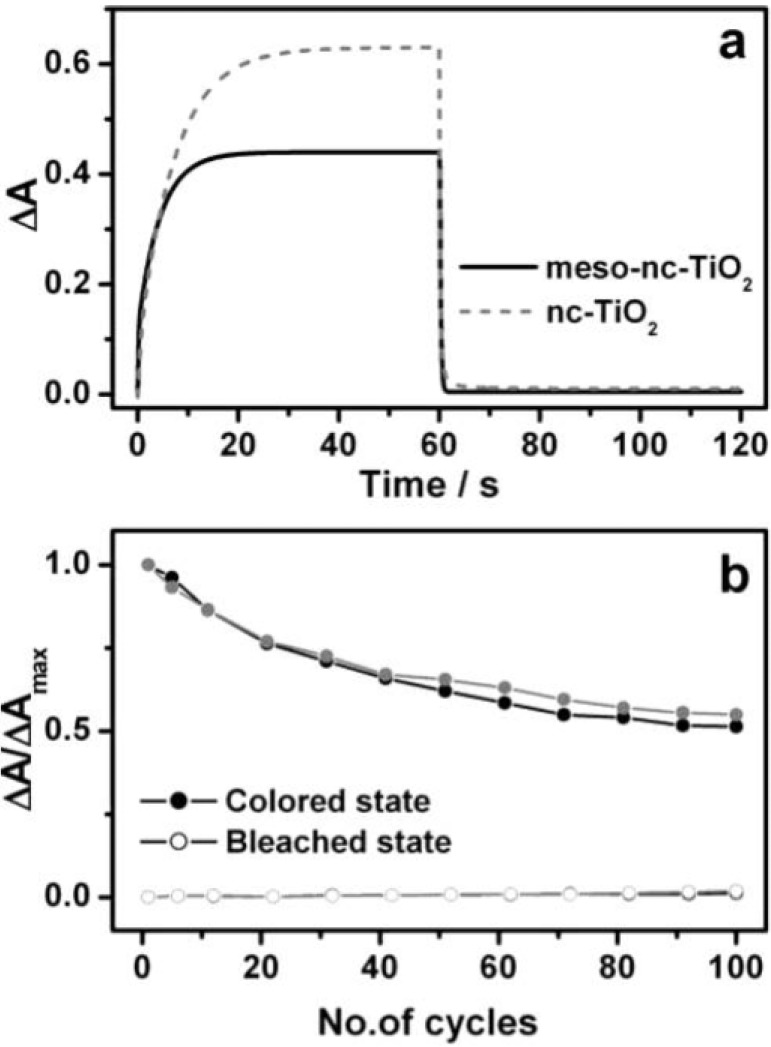
(a) Transient absorbance at 608 nm of the electrochromic cell made of viologen-modified mesoporous TiO_2_ and nanoscrystalline TiO_2_ electrodes following application of −2.5 V and 2.5 V, (b) relative absorbance profile of the colored state and bleached state of the electrochromic cells. Reproduced with permission from Ref. [50]; Copyright 2004 American Chemical Society.

Choi *et al.* [50] synthesized viologen modified mesoporous TiO_2_ film (Figure 17) with hexagonally close-packed mesopores and channel walls made of 8–10 nm anatase nanostructures. The film exhibited similar switching speed and reversibility as nanocrystalline titania but better contrast (Figure 18 and 19). The higher contrast can be attributed to a contiguous pathway of well-connected anatase nanocrystallites arranged into a well-defined mesoporous architecture, which results in a greater volume density of loaded viologen molecules.

**Figure 20:**
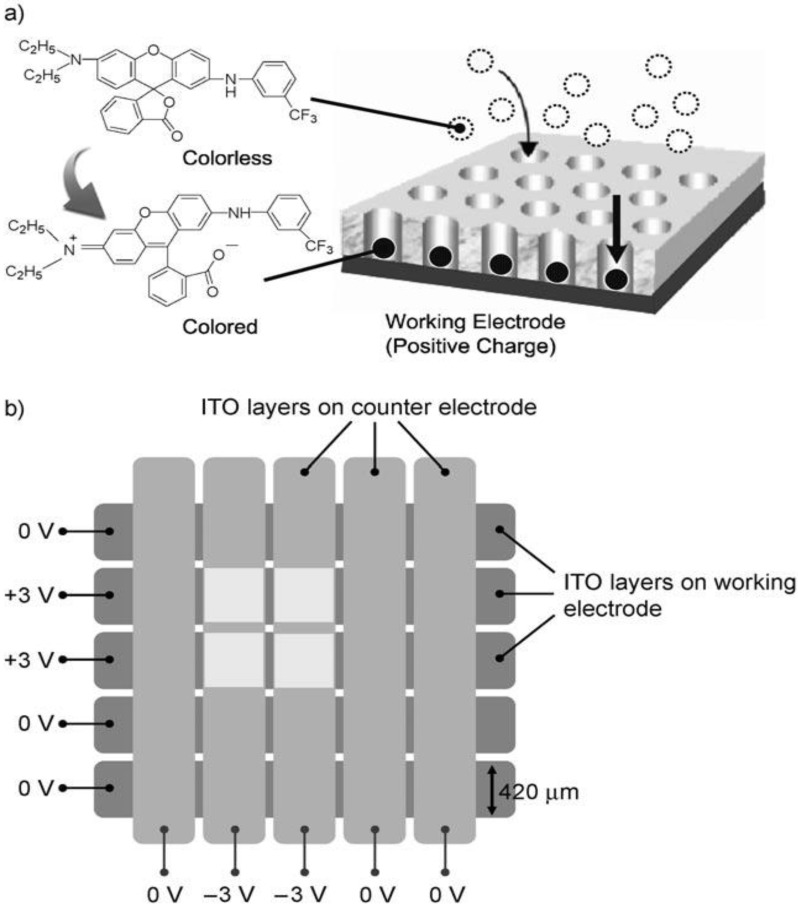
Illustration for (a) imaging using a leuco dye on a mesoporous TiO_2_ electrode with vertical pores and (b) imaging process for a passive-matrix electrochromic display. Reproduced with permission from Ref. [51]; Copyright 2010 Wiley-VCH Verlag GmbH & Co. KGaA.

Weng *et al*. [51] demonstrated a high-speed passive-matrix EC display using a leuco modified mesoporous TiO_2_ electrode with vertical porosity (Figure 20). The device exhibited better background whiteness, which improves readability and reduces eyestrain. Imaging and erasing can be carried out by applying a potential of ±3.0 V. The thickness of the mesoporous layer critically affects the contrast of the displayed image. Upon writing, the clear images can remain for a few minutes without becoming blurry because the vertical pores of the electrode can support effective diffusion of leuco dyes perpendicular to the electrode and prevent the diffusion of the dye around the electrode. The leuco modified mesoporous TiO_2_ electrode shows potential for the realization of a full-color reflective display for use in e-papers.

### 2.5. PEDOT Nanotubes

**Figure 21:**
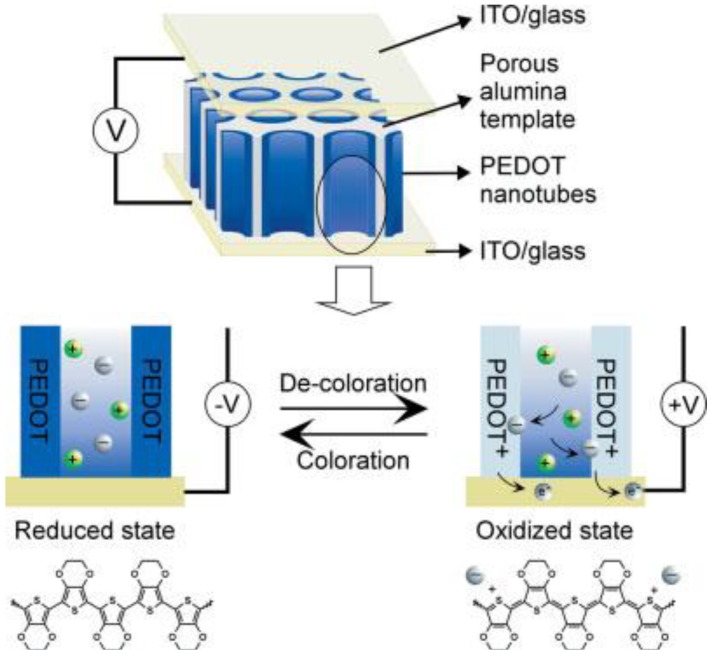
Schematic representation of the ultrafast EC device based on PEDOT nanotube arrays. Anions (negatively charged gray balls) diffuse into the thin wall (10–20 nm in wall thickness to provide the short diffusion distance) of the PEDOT nanotubes when PEDOT nanotubes are oxidized by applying a positive potential. The color of PEDOT turns from a deep blue to a transparent pale blue. Reproduced with permission from Ref. [52]; Copyright 2005 Wiley-VCH Verlag GmbH & Co. KGaA.

**Figure 22:**
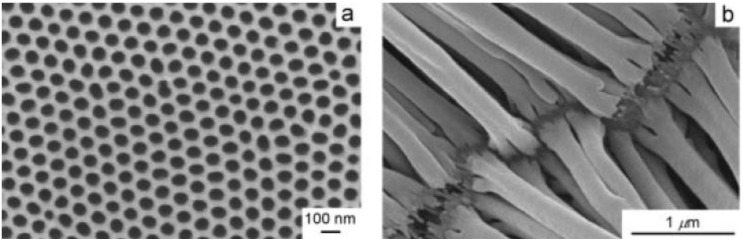
SEM images of (a) AAO template and (b) PEDOT nanotubes after dissolving the template. Reproduced with permission from Ref. [52]; Copyright 2005 Wiley-VCH Verlag GmbH & Co. KGaA.

PEDOT and its derivatives are good polymer EC materials for e-paper due to their high color contrast, good mechanical stabilities, and facile fabrication. Cho *et al*. [52] have synthesized PEDOT nanotubes using AAO as the template by electrochemical polymerization within the pores of the alumina films (Figure 21). The thin walls of PEDOT nanotubes (Figure 22b) can provide ions with short diffusion distances (10–20 nm), which allows very fast switching response, while the micrometer length of the PEDOT nanotubes can produce clear coloration contrast for the EC display system. The wall thickness of PEDOT nanotubes can be adjusted by controlling both the applied potential and the monomer concentration.

**Figure 23:**
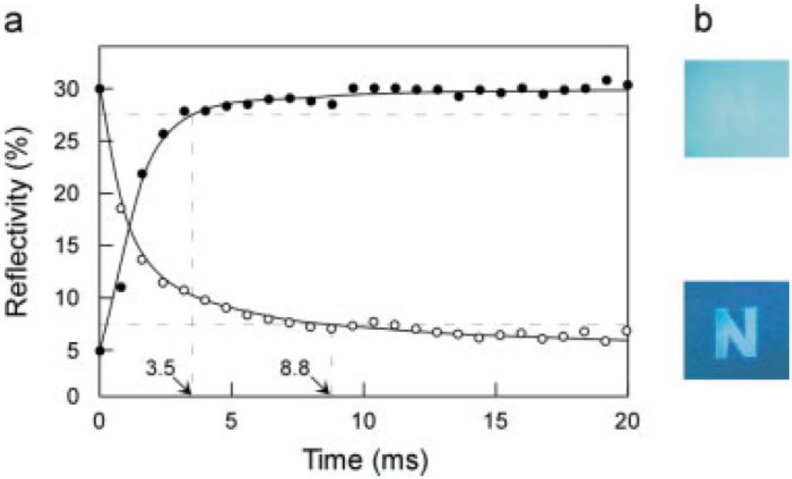
(a) Plots of reflectivity of EC window (1 cm^2^) monitored at 530 nm for coloration (open circles) and bleaching (solid circles) upon potential switching between −1.0 and 1.0 V, respectively. (b) Patterned letter “N” on the PEDOT nanotube arrayed film. Lower image: at −1.0 V, upper image: at 1.0 V. Reproduced with permission from Ref. [52]; Copyright 2005 Wiley-VCH Verlag GmbH & Co. KGaA.

The EC device can be switched from pale blue (bleached and oxidized state of PEDOT) to deep blue (colored and reduced state of PEDOT) by applying alternating square potentials between 1.0 V and −1.0 V (Figure 23). Both the coloration and bleaching processes show quite fast switching responses of less than 10 ms, which are fast enough for display applications. However, the contrast in this EC device is not high enough. To realize a practical application in EC display devices, the contrast still needs to be further enhanced.

### 2.6. V_2_O_5_ Nanowires

**Figure 24:**
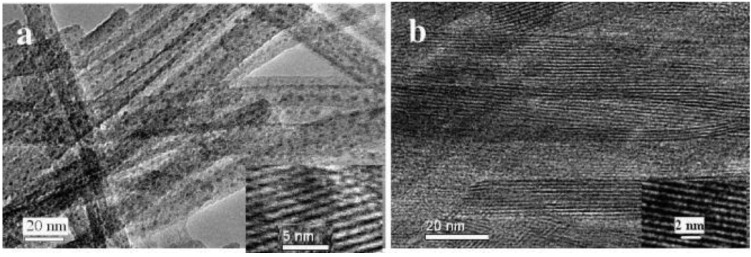
HRTEM images of (a) SVO nanowires and (b) V_2_O_5_ nanowires. Insets: images with high magnification. Reproduced with permission from Ref. [53]; Copyright 2008 American Chemical Society.

**Figure 25:**
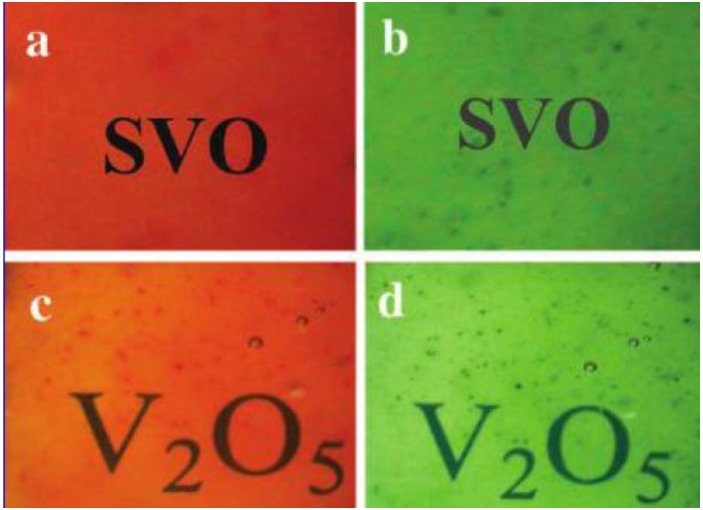
Digital photographs of (a) bleached, (b) colored states for a SVO nanowire-based EC device, (c) bleached and (d) colored states for a V_2_O_5_ nanowire-based EC device. Reproduced with permission from Ref. [53]; Copyright 2008 American Chemical Society.

**Figure 26:**
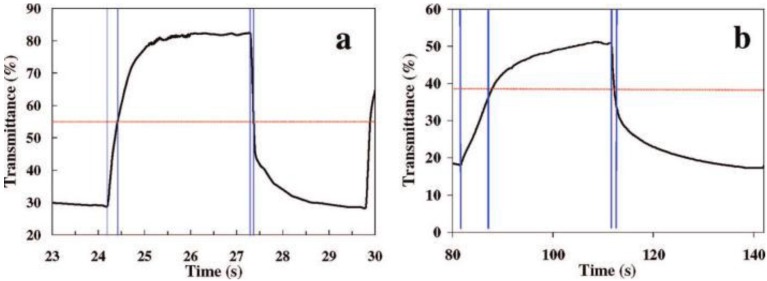
Transmittance *versus* time during coloration and bleaching cycles of (a) a SVO nanowire-based EC device and (b) a V_2_O_5_ nanowire-based EC device. Reproduced with permission from Ref. [53]; Copyright 2008 American Chemical Society.

Xiong *et al*. [53] prepared layered silver vanadium oxide (SVO) nanowires and V_2_O_5_ nanowires by hydrothermal process. The as-made nanowires are over 30 µm in length and 10–20 nm in diameter, corresponding to an aspect ratio of over 1500 (Figure 24). The nanowires with diameters of 10–20 nm have a short Li^+^ ions diffusion distance. The EC device displayed the coloration/bleaching between green (colored state) and red-brown (bleached state) (Figure 25). The coloration/bleaching switching responses for half of the total transmittance change, are about 0.1 s (coloration time) from red-brown to green and 0.2 s (bleaching time) for the reverse process at 633 nm (Figure 26a). For V_2_O_5_ nanowires, the coloration and bleaching times are 1 s from brown to green and 6 s for the reverse process (Figure 26b), respectively. The SVO nanowire-based EC device displayed color-switching responses over 20 times faster than those of the V_2_O_5_ nanowire-based EC device. These enhancements in EC properties of SVO nanowires should be attributable to their better electrical conductivity (0.5 S/cm) than V_2_O_5_ nanowires (0.08 S/cm) and the introduction of Ag atoms in the V_2_O_5_ framework further enlarged the interlayer spacing, resulting in a 7 times higher Li^+^ ions diffusion coefficient than that of V_2_O_5_ nanowires.

### 2.7. PB Nanoinks

PB, iron (III) hexacyanoferrate (II), a synthetic coordination-compounded pigment that has a three century history [54], is the prototype of a number of polynuclear transition metal hexacyanometallates which form an important class of insoluble mixed-valence compounds [55]. They have the general formula *M'*_k_ [*M''*(CN)_6_]*_l_* (*l*, *k* integral), where *M'* and *M''* are transition metals with different formal oxidation numbers with the possibility of change/electron transfer between their different oxidation states. For PB, the transition metal *M* is iron (Fe), *i.e*., ferric ferrocyanide.

In 1978, Neff [56] firstly prepared thin films of PB on platinum and gold electrodes and demonstrated the redox reactions accompanied by electrochromic behaviors. Since then, numerous investigations on the properties of PB films were prompted [57,58,59,60,61,62,63,64]. The electrochemical reduction and oxidation of PB can lead to the “Prussian white” (PW, Everitt’s Salt) and “Prussian green” (PG, Berlin green), respectively, and further oxidation of PG will lead to “Prussian yellow” [25].

**Figure 27:**
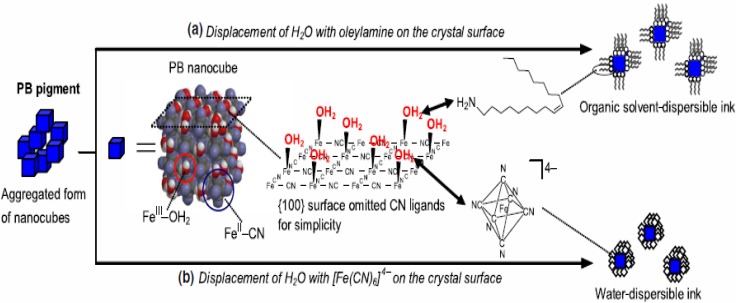
Schematic illustration of preparing PB nanoparticle inks from the insoluble PB pigment. Reproduced with permission from Ref. [70]; Copyright 2007 IOP Publishing Ltd.

**Figure 28:**
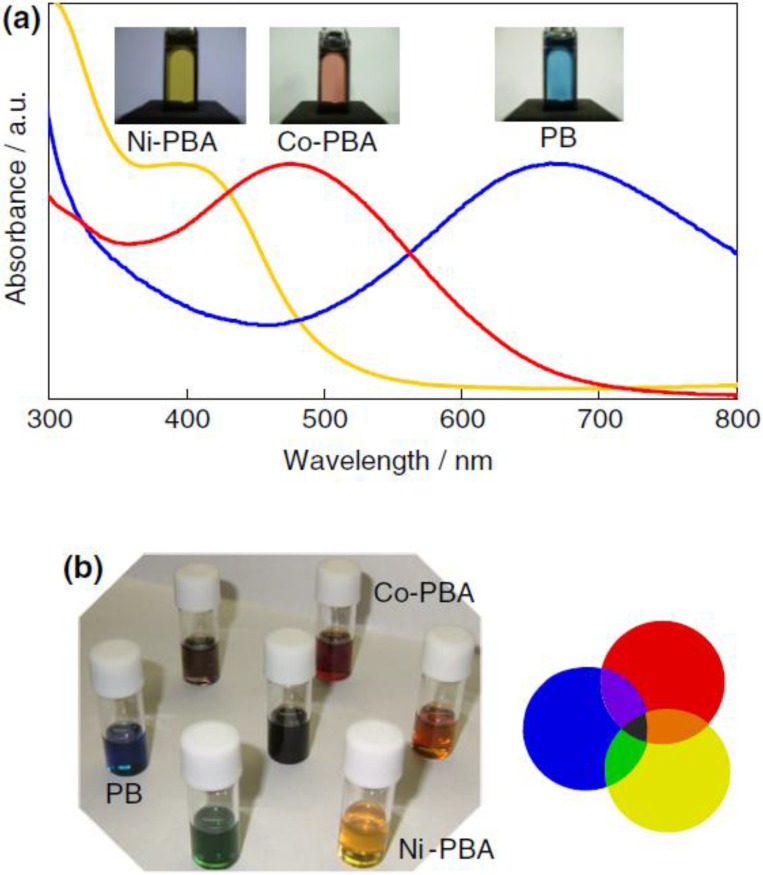
(a) UV-vis absorption spectra of the PB (blue line), Co-PBA (red line), and Ni-PBA (yellow line) nanoparticles in their transparent dilute toluene dispersion. (b) Hue circle produced by dichromatic tuning of the three inks. Reproduced with permission from Ref. [70]; Copyright 2007 IOP Publishing Ltd.

Generally, PB thin films are fabricated by an electrodeposition method through a bath containing iron (III) and hexacyanoferrate (ІІ) ions [65,66,67]. The detailed formation mechanism, corresponding reactions and chemical composition of electrodeposited PB films have been well summarized in previous reports [68,69]. Recently, water-soluble and organic-solvent-soluble PB nanoparticles inks have been successfully prepared by surface modification [70,71,72,73]. The strategies for the process are schematically shown in Figure 27. By surface modification of the insoluble PB pigment (agglomerated nanoparticles) with [Fe(CN)_6_]^4−^ ions and oleylamine, water-soluble and organic-solvent-soluble PB inks could be obtained, respectively. A similar ink preparation is also applicable to nickel hexacyanoferrates (Ni-PBA) and cobalt hexacyanoferrates (Co-PBA). The PB (blue), Ni-PBA (yellow), and Co-PBA (red) nanoinks function as the three primary colors for color displays and more colors can be obtained by mixing different nanoinks (Figure 28), which is an important progress for achieving full-color displays.

The soluble PB nanoinks can be uniformly spread on substrates, and PB films composed of aggregated nanoparticles can be fabricated after solvent evaporation. The EC thin films fabricated by spin-coating of PB nanoinks are usually not well-adhesive to transparent conductive substrates, resulting in elution from electrolytes. To overcome this difficulty, Omura *et al*. [74] successfully developed a method for controlling the elution using a simple electrochemical treatment. The PB nanoinks can be further patterned as desired by conventional lithography. Due to the solubility of the nanoinks, micro-fabrication processes, such as spin-coating, ink-jet printing and photolithography can be used in the fabrication of PB-based EC devices, which is cost-efficient, facile and easily scaled-up.

### 2.8. Nanostructures in Switchable Mirrors

Switchable mirrors can reversibly change their optical properties between reflective and transparent states as a result of hydrogenation and dehydrogenation [75,76,77,78,79]. Almost all heat and light can be reflected in their metal states, which is significant in energy-saving smart windows. Rare earth metals [80], magnesium lanthanide alloys [81], Mg-Ni alloys [82,83], Mg-Ti alloys [84] have been used as switchable mirror materials. However, rare earth metals are easily oxidized; Mg-Ni alloys are not color neutral; Mg-Ti alloys have low visible transmittance in their transparent states. Thin overlayers of palladium have been successfully used to protect the switchable mirrors against oxidation and enhance the kinetics of hydrogen insertion and extraction (acting as a catalyst for introduction and removal of hydrogen) [85,86].

Recently, Yamada *et al*. prepared switchable mirrors based on Mg-Ca alloy thin films by magnetron sputtering [87]. The switchable mirrors looked completely color neutral with high visible transmittance in the transparent state, which may be attributed to the grayish neutral hydrogenated state of MgH_2_ and MgCaH_3.72_.

Metallic copper exhibits very high near-infrared reflectance and much less reflectance below ~600 nm; Cu_2_O has low reflectance in both the visible and near-IR spectra. Metallic thin films of antimony and bismuth, deposited on glass substrates by vacuum evaporation, can be reversibly converted to transparent, semiconducting lithium pnictides by cathodic polarization in a nonaqueous lithium electrolyte. These characteristics make copper-copper (I) oxide, Sb-Li, Bi-Li [88] and Sb-Cu-Li [89] attractive in switchable mirrors.

## 3. Conclusions and Perspective

Since the discovery of EC materials, extensive investigations have been carried out to improve their EC properties, including enhancement of the switching responses; contrast; coloration efficiency and cycling stability; as well as EC device fabrications and applications. ZnO nanowire/nanorod arrays modified by EC materials, mesoporous and tubular EC materials significantly enhance the switching responses of EC devices, for potential application in fast-switching displays and e-papers. Crystalline WO_3_ nanoparticles, nanorods and crystalline mesoporous WO_3_ exhibit enhanced EC stability and contrast, especially in acidic electrolytes. Soluble PB and its analogues Ni-PBA, Co-PBA nanoinks exhibit blue, yellow and red colors, promising the possibility of achieving full-color displays. Due to the solubility of the nanoinks, micro-fabrication processes, such as spin-coating, ink-jet printing and photolithography, can be used in the fabrication of PB-based EC devices, which is cost-efficient, facile and easily scaled-up. Switchable mirrors are the most effective energy-saving EC devices in all kinds of smart windows. However, the metal alloys are easily oxidized, resulting in a poor long-term stability.

Further efforts are needed to accelerate practical applications of EC devices. For large-area EC devices, the difficulties may be in slow responses, poor stability and high cost. To overcome these difficulties, the synthesis of new nanostructured EC materials needs to be developed. The ideal nanostructures for EC materials may include ultrathin crystalline nanorods, nanowires or nanotubes, crystalline mesoporous structures and quantum dots. These nanostructures with large specific surface areas, are expected to possess fast and stable EC switching. Low-cost synthesis routes for these EC nanostructures are favorable. The coating process for EC nanostructures also needs to develop to obtain well-adhesive EC films on transparent conductive substrates. The combination of different kinds of EC materials is also worthwhile, for example, to exhibit multi-colors and to enhance the coloration modulation and stability. Complimentary EC devices need further development to enhance the contrast, coloration efficiency and stability of EC devices. Inorganic-organic hybrid EC devices are also promising for practical application, for example in e-papers. Besides the developments of EC materials and devices, transparent conductive substrates with better conductivity, solid-state electrolytes, advanced sealing and packaging technologies, are also critical and deserve intensive investigations for the practical applications of EC materials and devices. Gallium doped ZnO (GZO), antimony doped tin oxide (ATO), carbon nanotubes or graphene coated glass are promising substitutes to replace ITO. Low-cost processes for the fabrication of EC devices must be developed to realize their practical applications, which need the cooperation of researchers and technicians to solve both scientific and technical challenges.
